# Knowledge, Attitudes, and Practices of Nonpharmaceutical Interventions following School Dismissals during the 2009 Influenza A H1N1 Pandemic in Michigan, United States

**DOI:** 10.1371/journal.pone.0094290

**Published:** 2014-04-18

**Authors:** Jianrong Shi, Rashid Njai, Eden Wells, Jim Collins, Melinda Wilkins, Carrie Dooyema, Julie Sinclair, Hongjiang Gao, Jeanette J. Rainey

**Affiliations:** 1 Centers for Disease Control and Prevention, National Center for Emerging and Zoonotic Infectious Diseases, Division of Global Migration and Quarantine, Atlanta, Georgia, United States of America; 2 University of Michigan School of Public Health, Ann Arbor, Michigan, United States of America; 3 Michigan State University Program in Public Health, East Lansing, Michigan, United States of America; 4 Michigan Department of Community Health, Bureau of Epidemiology, Lansing, Michigan, United States of America; 5 Eagle Medical Services, San Antonio, Texas, United States of America; University of California, United States of America

## Abstract

**Background:**

Many schools throughout the United States reported an increase in dismissals due to the 2009 influenza A H1N1 pandemic (pH1N1). During the fall months of 2009, more than 567 school dismissals were reported from the state of Michigan. In December 2009, the Michigan Department of Community Health, in collaboration with the United States Centers for Disease Control and Prevention, conducted a survey to describe the knowledge, attitudes, and practices (KAPs) of households with school-aged children and classroom teachers regarding the recommended use of nonpharmaceutical interventions (NPIs) to slow the spread of influenza.

**Methods:**

A random sample of eight elementary schools (kindergarten through 5^th^ grade) was selected from each of the eight public health preparedness regions in the state. Within each selected school, a single classroom was randomly identified from each grade (K-5), and household caregivers of the classroom students and their respective teachers were asked to participate in the survey.

**Results:**

In total, 26% (2,188/8,280) of household caregivers and 45% (163/360) of teachers from 48 schools (of the 64 sampled) responded to the survey. Of the 48 participating schools, 27% (13) experienced a school dismissal during the 2009 fall term. Eighty-seven percent (1,806/2,082) of caregivers and 80% (122/152) of teachers thought that the 2009 influenza A H1N1 pandemic was severe, and >90% of both groups indicated that they told their children/students to use NPIs, such as washing hands more often and covering coughs with tissues, to prevent infection with influenza.

**Conclusions:**

Knowledge and instruction on the use of NPIs appeared to be high among household caregivers and teachers responding to the survey. Nevertheless, public health officials should continue to explain the public health rationale for NPIs to reduce pandemic influenza. Ensuring this information is communicated to household caregivers and teachers through trusted sources is essential.

## Background

The 2009 influenza A H1N1 pandemic (pH1N1) in North America affected school-aged children more than adults and resulted in school outbreaks during the spring 2009 pandemic wave [1], [2]. The United States Centers for Disease Control and Prevention (CDC) initially recommended school dismissals (closing schools for student education) for 7 days for kindergarten through grade 12 (K-12) schools in an effort to mitigate influenza transmission [3]. However, federal nonpharmaceutical intervention (NPI) guidance was subsequently updated to recommend that students and/or staff stay home until fever free for 24 hours if ill with influenza-like illness, unless student or staff absenteeism reached a level that interfered with the school's ability to function, in which case, schools could be reactively dismissed [4]. The pandemic impacted the state of Michigan in two waves, with the highest peak of influenza activity occurring in the second wave from October to November 2009 [5]. During this second fall wave, Michigan experienced a large number of school dismissals. Even though the Michigan Department of Education (MDE) did not have a closure policy based on absenteeism, these reactive school dismissals primarily occurred following an abrupt rise in school absenteeism, beginning the third week of October, 2009. Student absenteeism rates exceeded 25% at the time of most school dismissals, with dismissals ranging in duration from one to eight school days [6]. By November 30, 2009, 567 schools in Michigan had been dismissed for ≥1 day as a result of pH1N1 [6], more school dismissals than any other state.

In December 2009, the Michigan Department of Community Health (MDCH), in collaboration with CDC, conducted a household caregiver and school teacher survey regarding their knowledge, attitudes, and practices (KAPs) about pH1N1 as well as their instruction on the use of recommended NPIs to slow the transmission of influenza. The purpose of the survey was to assess the impact of school dismissals on households' and teachers' influenza KAPs and NPI use. Information derived from the survey can assist school administrators and public health officials to improve preparedness activities and the decision-making process, including communicating the public health threat and the rationale of school dismissals and NPI recommendations, during a future influenza pandemic.

## Methods

### Survey population

At the time of the survey, Michigan was divided into eight public health preparedness regions (PHPRs), and 957 public and private elementary schools operated across these regions [6]. In Michigan, elementary schools go from kindergarten to the 5th grade (for students approximately 5 to 10 years of age). A random sample of eight elementary schools was selected from each of the eight PHPRs, for a sample of 64 schools. Within each school, a single classroom was randomly identified from each grade (K-5), and teachers and students' caregivers were asked to participate in the survey. Investigators sent an email letter regarding the survey to local health departments, superintendents, and principals of selected elementary schools. The letter described the purpose of the survey, information regarding the selected classrooms, and instruction for completing the survey.

### Ethics

The United States Centers for Disease Control and Prevention (CDC) and Michigan Department of Community Health (MDCH) Human Subjects Coordinators reviewed the study protocol, the questionnaire, and all other study materials and determined that the study did not constitute human subjects research and therefore was exempt from federal requirements for institutional review board consideration.

### Survey instrument

We developed three questionnaires for the survey. Two household questionnaires were distributed to caregivers: one for caregivers of students who attended a school that was dismissed during the fall 2009, and the other for caregivers of students who attended a school that remained open throughout the fall term. The household questionnaires collected information regarding household KAPs associated with pH1N1, instruction on the use of NPIs, and public health messaging (e.g., from media sources, health care providers, and school officials) about the pandemic. Questionnaires were distributed as a ‘backpack’ survey (students were given a hardcopy of the survey at school that was to be completed by the caregiver at home and later returned to the school by the student). The third questionnaire was distributed to teachers in the selected classrooms, and collected information about their KAPs and instruction on NPI use as well as methods used for communicating information to students. The first page of the survey stated the purpose of the investigation and noted that response was completely voluntary. We obtained data regarding school dismissals, attendance records, and demographic and community characteristics from MDCH and MDE.

### Data analysis

Survey data were entered into an Access database and exported to SAS 9.3 and JMP 10 (Cary, NC) for analysis. We calculated survey participation rates and generated descriptive statistics to summarize KAPs regarding the pandemic and instruction for NPI use from both household and teacher responses. We examined differences between household and teacher responses as well as differences between households with children at schools that were dismissed and those with children at schools that remained open. The survey compared responses according to level of concern regarding the pandemic. We used Pearson's Chi-square tests to examine differences between groups. A significance level of 5% was applied in the data analysis. Because 23 hypotheses were tested for caregiver and teacher responses comparing closed and open schools, a Bonferroni-adjusted significance level of 0.0022 was calculated to account for the increased possibility of a type-I error. For these comparisons, differences were considered statistically significant if p<0.0022.

## Results

**Figure 1:**
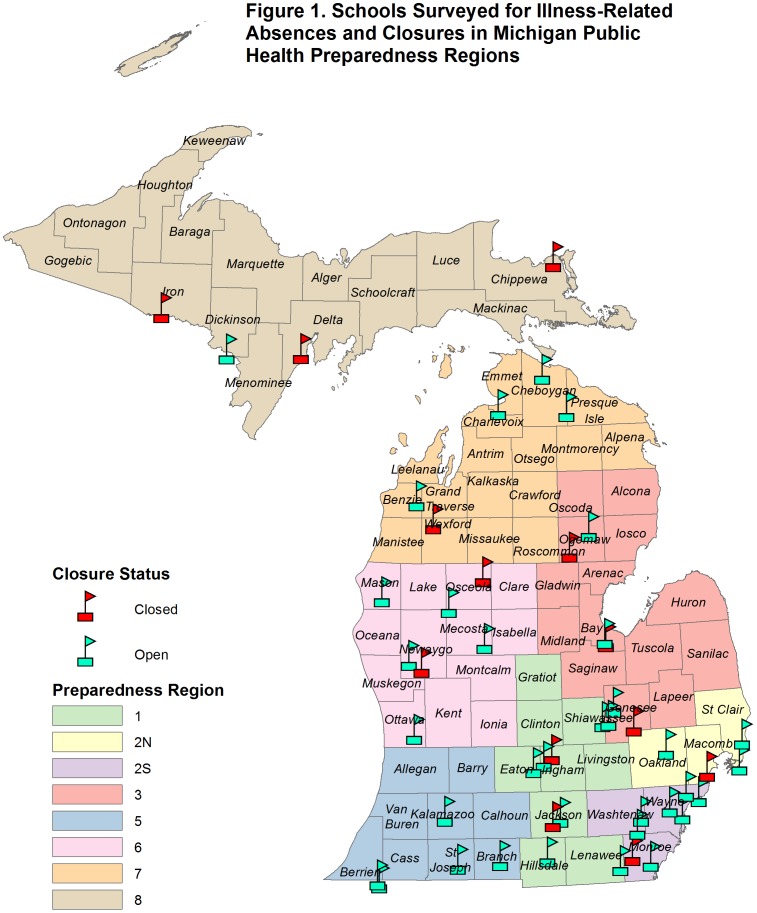
Schools Surveyed for Illness-Related Absences and Closures in Michigan Public Health Preparedness Regions.

Overall, 26% (2,188/8,280) of household caregivers and 45% (163/360) of teachers from 48 schools (of the 64 randomly selected) in the eight PHPRs responded to the survey [Figure 1]. Of the 48 participating schools, 27% (13) reported a school dismissal, while 73% (35) remained open during the 2009 fall term.

### Knowledge, attitudes, and practices (KAPs) of pH1N1 and school dismissals

Most (87%) caregivers thought pH1N1 was somewhat severe, severe, or very severe, and 80% of teachers provided the same response (Table 1). Sixty-two percent of caregivers and 52% of teachers were concerned that they, their children, or the students may get sick from influenza, and most caregivers (88%) and teachers (81%) thought excessive student absenteeism was the major reason for school dismissals. At the same time, 92% of caregivers and 89% of teachers reported that they believed school dismissals were somewhat effective or very effective in reducing influenza cases among school-aged children.

**Table 1:** Household caregiver and teacher responses to school dismissal survey* on knowledge, attitudes, and practices regarding pH1N1.

Knowledge, attitudes, and practices (KAPs)	Caregivers (n = 2188)	%	Teachers (n = 163)	%	p-Value‡
Response rate	2188/8280	26.4	163/360	45.3	
Felt H1N1 Flu was severe	1806/2082	86.7	122/152	80.3	0.025
Concerned about being sick from flu	1182/1893	62.4	72/138	52.2	0.017
Felt school closure was effective†	503/546	92.1	122/137	89.1	0.249
Major reasons for school closure†					
To clean the building	377/535	70.5	29/52	55.8	0.029
To keep children apart	468/561	83.4	45/53	84.9	0.781
Many students absent from school	515/586	87.9	42/52	80.8	0.140
Many teachers absent from school	102/432	23.6	10/47	21.3	0.720

* Schools selected randomly (irrespective of size) from each of the eight public health preparedness regions in Michigan, United States.

† Households attending dismissed schools.

‡ Pearson's Chi-square test was used to compare differences between caregivers and teachers.

Differences were considered statistically significant if p<0.05.

### Instruction on the use of nonpharmaceutical interventions (NPIs)

Eighty-one percent of caregivers and 65% of teachers reported telling their children or the students to stay away from sick people (Table 2). More than 90% of caregivers and teachers told their children or the students to wash their hands more often, use hand sanitizer, cough or sneeze in their elbow, cover coughs with tissues, and avoid sharing drinks. Among those surveyed, 43% of caregivers and 13% of teachers also told their children or the students to avoid crowds as a means to lower the chance of contracting influenza. We also analyzed instruction for NPI use according to whether caregivers and teachers were concerned about being infected with pH1N1. Caregivers who reported concern were more likely than non-concerned caregivers to instruct their children to use NPIs, including staying away from sick people, washing hands more often, using hand sanitizer, coughing or sneezing in their elbow, avoiding sharing drinks and avoiding crowds (*p*<0.05).

**Table 2:** Household caregiver and teacher instruction to children/students on the use of nonpharmaceutical interventions (NPIs), by concern about being infected with influenza H1N1.

Instructions to children/students on the use of nonpharmaceutical interventions (NPIs)	Caregivers (n = 2188)				Teachers (n = 161)				
	Total	Concerned	Not concerned	p-Value*	Total	Concerned	Not concerned	p-Value*	p-Value†
	(n = 1893)	(n = 1182)	(n = 711)		(n = 136)	(n = 71)	(n = 65)		
	(%)	(%)	(%)		(%)	(%)	(%)		
Stay away from sick people	1472/1811	984/1137	488/674	<0.001	81/124	45/64	36/60	0.228	<0.001
	(81.3)	(86.5)	(72.4)		(65.3)	(70.3)	(60.0)		
Wash hands more often	1836/1870	1156/1168	680/702	0.001	132/134	69/70	63/64	0.949	0.784
	(98.2)	(99.0)	(96.9)		(98.5)	(98.6)	(98.4)		
Cover cough with tissues	1772/1867	1110/1164	662/703	0.256	121/133	63/70	58/63	0.678	0.051
	(94.9)	(95.4)	(94.2)		(91.0)	(90.0)	(92.1)		
Use hand sanitizer	1674/1786	1062/1116	612/670	0.001	122/133	65/69	57/64	0.282	0.364
	(93.7)	(95.2)	(91.3)		(91.7)	(94.2)	(89.1)		
Cough or sneeze in their elbow	1787/1859	1128/1162	659/697	0.006	129/134	67/70	62/64	0.723	0.935
	(96.1)	(97.1)	(94.5)		(96.3)	(95.7)	(96.9)		
Avoid sharing drinks	1723/1838	1100/1150	623/688	<0.001	117/129	62/68	55/61	0.843	0.174
	(93.7)	(95.7)	(90.6)		(90.7)	(91.2)	(90.2)		
Avoid crowds	747/1756	551/1105	196/651	<0.001	15/120	11/64	4/56	0.097	<0.001
	(42.5)	(49.9)	(30.1)		(12.5)	(17.2)	(7.1)		

* Pearson's Chi-square test was used to compare NPIs between caregivers who reported concern and non-concerned caregivers.

† Pearson's Chi-square test was used to compare NPIs between caregivers and teachers.

Differences were considered statistically significant if p<0.05.

### Impact of school dismissals on the use of nonpharmaceutical interventions (NPIs)

In response to pH1N1, 83% of caregivers reported that their children had taken steps to avoid being near someone with influenza like illness in both dismissed and open schools. Between 11%–36% of caregivers also reported going to fewer places such as movies, sporting events or concerts, avoiding crowds, going to large shopping areas or malls less often, using public transportation (e.g., buses and trains) less often, avoiding events such as parties, wedding ceremonies or family gatherings, going to church, temple, mosque or other places of worship less often, and keeping children home because classmates were sick (Table 3). These behaviors were statistically similar across households impacted by schools dismissals and households that were not (*p*>0.0022). On the other hand, teachers at schools experiencing a pandemic-related dismissal were more likely to support cancelations or the re-scheduling of after-school activities (67%), sport practices and games (68%), school performances (36%), and school field trips (40%) compared to teachers at schools that remained open during the fall 2009 (*p*<0.001). There was no statistically significant difference in cleaning the school, promotion of personal protective measures or use of other social distancing measures between dismissed and open schools according to the teachers' survey.

**Table 3:** Household caregiver and teacher responses to selected items from school dismissal survey on nonpharmaceutical interventions (NPIs) by school dismissal Status in fall 2009*, from 48 elementary schools†, Michigan, USA.

Nonpharmaceutical interventions (NPIs)			School closure				
	Total	%	Yes	%	No	%	p-Value‡
Caregivers (Response rate)	2188/8280	26.4	659/2484	26.5	1529/5796	26.4	0.888
In response to flu, a family has done any of the following since September 2009							
Gone to fewer places like movies, sporting events or concerts	703/1987	35.4	212/576	36.8	491/1411	34.8	0.396
Gone less often to large shopping areas or malls	679/1999	34.0	187/580	32.2	492/1419	34.7	0.298
Used public transportation such as buses and trains less often	468/1713	27.3	115/486	23.7	353/1227	28.8	0.033
Avoid events such as parties, wedding ceremonies or family gatherings	259/1997	13.0	76/584	13.0	183/1413	13.0	0.970
Gone less often to church, temple, mosque or other places of worship	229/1955	11.7	59/570	10.4	170/1385	12.3	0.229
Taken any steps to avoid being near someone with flu-like symptoms	1686/2043	82.5	486/593	82.0	1200/1450	82.8	0.665
Avoid crowds	708/1996	35.5	192/576	33.3	516/1420	36.3	0.204
Kept children home because classmates were sick	214/2006	10.7	61/584	10.4	153/1422	10.8	0.836
Did not send children to aftercare or childcare	242/1780	13.6	65/502	12.9	177/1278	13.8	0.618
Activities were changed because of the flu							
After school activities	296/1408	21.0	213/317	67.2	83/1091	7.6	<0.001
Sports practices and games	225/1292	17.4	175/281	62.3	50/1011	4.9	<0.001
School performances	102/1295	7.9	86/213	40.4	16/1082	1.5	<0.001
School field trips	141/1370	10.3	112/231	48.5	29/1139	2.5	<0.001
Teachers (Response rate)	163/360	45.3	38/108	35.2	125/252	49.6	0.012
Teacher was doing any of following things to prevent kids from getting the flu							
Cleaning the school more often	118/163	72.4	28/38	73.7	90/125	72.0	0.839
Encouraging students to cover their cough and sneeze	139/163	85.3	31/38	81.6	108/125	86.4	0.463
Encouraging students to wash their hands	137/163	84.0	30/38	78.9	107/125	85.6	0.327
Providing hand sanitizers in the classrooms or hallways	100/163	61.3	24/38	63.2	76/125	60.8	0.794
Recommending that students with flu-like illness stay home at least 24 hours	74/163	45.4	15/38	39.5	59/125	47.2	0.402
Rearranging the classroom to keep kids further apart	23/163	14.1	5/38	13.2	18/125	14.4	0.847
Activities were changed because of the flu							
After school activities	18/115	15.7	16/24	66.7	2/91	2.2	<0.001
Sports practices and games	16/104	15.4	15/22	68.2	1/82	1.2	<0.001
School performances	6/104	5.8	5/14	35.7	1/90	1.1	<0.001
School field trips	8/113	7.1	6/15	40.0	2/98	2.0	<0.001

* Schools selected randomly (irrespective of size) from each of the eight public health preparedness regions in Michigan.

† Of the 48 schools included in the survey, 13 were reactively dismissed due to student/teacher absenteeism and 35 remained open during the fall 2009.

‡ p-Values are calculated based on Pearson's Chi-square test.

Differences were considered statistically significant if p<0.0022.

### Communicating with households and teachers

Overall, 94% (141/150) of surveyed teachers indicated that their schools provided information to parents regarding the pandemic during the fall 2009 school term. Of the responding household caregivers, 83% felt that they received enough information (letter, email, and flyer) from their child's school about the flu in the 2009 school year. Caregivers also actively sought information from other sources, such as the CDC, state or local public health departments, TV, newspapers, their doctors, and the internet (Table 4). In addition to obtaining information from public health and medical care staff, 3% (55/2,188) of participants reported obtaining at least some information about pH1N1 from online social media (Twitter, blogs, Facebook, or discussion boards). There was no statistically significant difference in the source of information for households attending dismissed schools or schools that remained open (*p*>0.05).

**Table 4:** Household caregiver responses regarding source of information on the 2009 influenza A H1N1 pandemic (pH1N1)*.

			School closure				
Questions about sources of information household caregivers sought out in regards to the spread of flu	Total (n = 2188)	%	Yes (n = 659)	%	No (n = 1529)	%	p-Value†
Do you feel you received enough information (letter, email, flyer, etc.) from your child's school about the flu this school year (September, 2009)?	1745/2090	83.5	509/609	83.6	1236/1481	83.5	0.945
Have you sought out any of the following sources in regards to the spread of flu or school closure status?	1274/2188	58.2	349/659	53.0	925/1529	60.5	0.065
Government health officials‡	646/2188	29.5	176/659	26.7	470/1529	30.7	
Schools§	627/2188	28.7	160/659	24.3	467/1529	30.5	
Media (TV, Radio or Newspaper)	853/2188	39.0	230/659	34.9	623/1529	40.7	
Online social media (Twitter, blogs, Facebook or discussion boards)	55/2188	2.5	17/659	2.6	38/1529	2.5	
Others (Doctors, nurses, and phone calls)	133/2188	6.1	21/659	3.2	112/1529	7.3	

* Schools selected randomly (irrespective of size) from each of the eight public health preparedness regions in Michigan.

† p-Values are calculated based on Pearson's Chi-square test.

‡ Government Health Officials include the U.S. Centers for Disease Control and Prevention as well as state and local health departments in Michigan.

§ Schools include teachers, principals, superintendents, school staff, PTA (Parent and Teacher Association) and school websites.

## Discussion

This survey describes households' and teachers' knowledge, attitudes, practices (KAPs) and their instruction on the use of nonpharmaceutical interventions (NPIs) during the fall 2009 influenza A H1N1 pandemic (pH1N1) and whether school dismissals influenced their perception of pH1N1 and NPI use. CDC recommended the increased use of NPIs in school settings during the 2009 H1N1 influenza pandemic [4]. “Wash your hands, cover your cough, and stay home from school or work when you are sick” were the prevailing messages throughout the 2009 H1N1 pandemic [5]. Knowledge regarding pH1N1 and instruction for NPI use were high among caregivers and teachers in surveyed elementary schools. Instruction for increasing NPI use was associated with having a concern or fear regarding the pandemic, which was similar for dismissed schools and schools that remained open. The similarity regarding the concern among caregivers across all schools could reflect marginal differences in illness rates among students and teachers at dismissed and open schools as well as access to similar media information about the pandemic. In other regions impacted by the pandemic, households with good knowledge of pH1N1 were also more likely to comply with NPI recommendations compared to those with only a limited knowledge about the pandemic [7]. In Melbourne, Australia, for example, household-level compliance with restrictions on social outings was high, primarily due to heightened public awareness of the newly introduced pH1N1 virus of uncertain severity [8].

Our survey found that most caregivers and teachers believed that the school dismissals implemented in Michigan would reduce the number of influenza cases among children attending schools. However, reactive dismissals typically occur when transmission of influenza-like-illness is already wide spread in the community and are unlikely to affect community-wide transmission during a pandemic [9]. Dismissals in Michigan were for the most part reactive, and occurred after an abrupt increase in student or staff absenteeism. The MDE did not have a closure policy based on absenteeism levels during the 2009 pandemic; however, absenteeism exceeded 25% at the time of most school dismissals [5], so maintaining normal school activities was not possible. Dismissals in Michigan were short-lived, lasting an average of 4.7 days (range of 1–8 days) [6]. Similar to previous influenza outbreak-related school dismissals in Illinois, Kentucky, and North Carolina [10]–[12], Michigan school dismissals were generally accepted by caregivers, even if implemented reactively because of high absentee levels. This finding was also consistent with information from a public opinion poll conducted early in the pandemic, which suggested that families would support school dismissals if implemented [13].

As opposed to reactive school dismissals, pre-emptive dismissals (implemented prior to widespread disease in the community) are intended to slow transmission during an influenza pandemic by decreasing social mixing between school-aged children [14]–[16]. For example, in the greater Mexico City area, pH1N1 transmission decreased 29%–37% following pre-emptive school dismissals and implementation of other social distancing measures [17]. Similarly, the 2009 school summer break in Alberta, Canada was associated with a 50% reduction in influenza transmission among school-aged children, after adjusting for climate factors [18]. Pre-emptive school dismissals were implemented in very few circumstances in Michigan, primarily due to decisions that were not mediated by MDCH/CDC recommendations. Pre-emptive school dismissals, however, can involve costs and secondary consequences, and such dismissals will most likely be implemented during pandemics associated with high levels of moderate to severe disease and influenza related mortality [9]. In these scenarios, such as during the 1918 pandemic, the reduction in influenza morbidity and mortality is likely to outweigh the consequences associated with school dismissals [9]. In less severe pandemics, similar to pH1N1, secondary consequences of a pre-emptive closure may be too high, and the majority of school dismissals, such as in Michigan, will likely be implemented reactively as a result of high or increasing rates of illness, absenteeism, or public concern [9].

Reactive school dismissals did not appear to impact NPI use. Most caregivers and teachers among dismissed schools and schools that remained open recommended that children use NPIs as often as possible. A school-based intervention to increase the use of NPIs mitigated an influenza outbreak in a large school system in New York City [19]. Similar strategies were implemented elsewhere, including in most schools in Georgia [20] and Pennsylvania [21]. A previous study that showed that children can quickly learn and implement NPIs suggests the potential benefits of these recommendations [22]. In addition, most caregivers with children in both dismissed schools and schools that remained opened reported encouraging their children to avoid contact with ill classmates and others to lower the chance of contracting influenza. Compared with schools that remained open, caregivers of children in dismissed schools reported slightly less use of public transportation, and administrators more frequently postponed or cancelled after school activities. Both of these findings can be expected during school dismissals since demand for transportation to and from school and school-related activities is decreased.

A number of information sources were available to households regarding pH1N1. Although most caregivers actively sought information from the CDC, state or local public health departments, TV, newspapers and their doctors. Some caregivers also reported obtaining information from online social media (Twitter, Facebook and blogs). Health departments should be aware of these new sources, and ensure that accurate information is provided whenever possible. We were unable to assess the impact of each information source on the knowledge and instruction on the use of NPIs because household respondents accessed several different sources during the pandemic.

This survey is subject to a number of limitations. First, we relied on 48 of the 64 sampled schools across the eight public health preparedness regions (PHPRs) in the state. Though we attempted to include an equal number of schools from each of the eight PHPRs, our rapid sampling method did not adjust for school enrolment and, as such, a small school had the same selection probability as a larger school. Schools in urban areas could have been under-represented, which would limit the representativeness of our findings. Additionally, we could not assess whether survey respondents were demographically similar to the targeted population of households with children at the 64 selected schools. However, our findings are similar to those obtained from similar studies conducted elsewhere during the fall wave, and therefore likely reflect the general knowledge of the pandemic and instruction for NPI use at the time of the survey. Second, our response rates were low with only 26% of households and 45% of teachers responded to the survey. We anticipate that the higher response rates of teachers reflect that the surveys were distributed directly to school principals. Third, our survey instrument did not request information on household influenza-like-illnesses (ILI) and we were unable to link our school-based survey to sentinel influenza surveillance sites to assess whether instruction for NPI use was associated with changes in disease reports. Future projects aimed at capturing self-reported ILI data or by new methodological approaches could assist in evaluating the effectiveness of NPIs in mitigating influenza and other acute respiratory infections, especially in the school setting. Finally, recall bias could have influenced responses. By conducting the survey during December 2009 and referencing events occurred during the preceding two months, we likely limited this potential bias. Nevertheless, we should note that health behavior, including adherence to NPIs, could have changed during the course of the pandemic. Though transmission rates remained high, changes in perception about the threat of pH1N1 during the later phases of the second wave could have impacted the number and percentage of households continuing to instruct their children to use NPIs. This is supported by the fact that in our survey NPI instruction was greatest among household caregivers reporting that the pandemic was somewhat severe or severe.

## Conclusions

Reactive school dismissals due to excessive student absenteeism were implemented in a large number of schools in Michigan. Knowledge and instruction on the use of NPIs were high among caregivers and teachers but were linked primarily to perceived concerns regarding pH1N1. Public health officials should continue to explain the public health rationale for NPIs, including the benefits and limitations of pre-emptive versus reactive school dismissals and when pre-emptive and reactive measures are best used. Ensuring this information is communicated to household caregivers and classroom teachers through trusted sources is essential.
